# The Vapour-Vapour Interface Observation and Appraisement of a Gas-Condensate/Supercritical CO_2_ System

**DOI:** 10.1038/s41598-018-32622-9

**Published:** 2018-09-28

**Authors:** Ying Jia, Yunqing Shi, Lei Huang, Jin Yan, Rongchen Zheng, Lei Sun

**Affiliations:** 1 Exploration and Production Research Institute, Sinopec, Beijing 100083 China; 2 Research Institute of Petroleum Exploration and Development, PetroChina, Beijing 100083 China; 3 South West Petroleum University, Chengdu, 610500 China

## Abstract

Injecting supercritical CO_2_ into gas reservoir is a novel trial to improve condensate gas recovery and decrease the hydrocarbon liquid dropout. A good understanding of the effect of supercritical CO_2_ on the phase behavior properties of these hydrocarbons is essential for accurately forecasting the displacing performance and storing process of the reservoirs with numerical simulators. This paper presents novel phase behavior experimental procedures and phase equilibrium evaluation methodology for gas-condensate phase system mixed with supercritical CO_2_ over a wide range of temperatures and pressures. A unique phase behavior phenomena was also reported. The mass transfer between two vapour phases was also measured. In order to interpret and identify the interface property between condensate gas and supercritical CO_2_, a multiphase thermodynamic VLV equilibrium model was established. Finally, taken YKL condensate gas in Northwest China as an example, the region where the conditions in terms of pressure, temperature and CO_2_ concentration can yield VLV equilibrium was found. The calculation results of multiphase thermodynamic model for condensate-CO_2_ system in this paper are close to the experimental data and can truthfully reflect the phase behavior of interface between CO_2_ and condensate gas. The research results indicate that it is the existence of the interface between CO_2_ and condensate gas that makes CO_2_ possible be an attractive option to successfully displace condensate gas and decrease CO_2_ emissions.

## Introduction

Today, there is a wider energy mix that allow us to meet the energy demand however fossil fuels are the leading source of energy. Statistics show that natural gas currently provides approximately a quarter of the world’s energy and its share is increasing significantly^1^. The rapid increase in the worldwide demand of natural gas has resulted in a significant growth of international gas trade and stimulated long-term contracts for its sales. Hence, it becomes important to extend the lifetime of these reservoirs and meet the desired production rates.

Generally, natural gas resources are mainly distributed in dry gas and condensate gas reservoirs. As an essential part of Sinopec (China Petroleum & Chemical Corporation) in China’s hydrocarbon resources, the recovery of condensate gas is still low (Table 1). Currently, most of the condensate gas resources that have been discovered in China are distributed in Xinjiang province and offshore, of which saturated condensate content are middle-low^2^. The typical development method is under primary depletion. However, the recovery of condensate gas is only around 40% and some reservoirs have entered middle/late development periods. It is necessary to explore new techniques to improve condensate gas recovery.

**Table 1:** The statistics of recovery of different condensate gas fields^2,31–44^.

Gas field	Country	Geological Structure	Lithology	porosity (%)	permeability (mD)	Gas recovery (%)	Oil Recovery (%)	Development method
QL.	Russia	Anticline	sandstone	6–24	8.8–391.84	95		Depletion
Kov.	Russia		sandstone	5.4	1–100	65		Depletion
Min.	Russia	Anticline	sandstone	11.3	0.098–34.1	77.9		Depletion
Б yrra-m-ope	Azerbaijan	Anticline	sandstone	14–18	0.03	78.05	33.38	Depletion
J-E-B	USA	Faults and conformity trap	sandstone	27.4–33.6	200–1800	80	50	Cyclic Injection
N. Geragai G40	India	Dome	sandstone	20–27.5	18–900			Depletion
BZBII	China	Anticline	sandstone	23.4	240.63	48		Depletion
YH	China	Anticline	sandstone	15.4	51.1	67	54.7	Cyclic injection
KKY	China	Anticline	sandstone	9–18	5.5–126	59	39.2	Cyclic injection
QK (Sinopec)	China	Fault Block	sandstone	10.8	1.1	9.01		Depletion
BM (Sinopec)	China	Fault Block	sandstone	11	0.1–6	22.22		Depletion
YK (Sinopec)	China	Anticline	sandstone	12.5	88.7	43	37.92	Depletion

The low recovery is due to complex physical and chemical phase transitions in condensate gas reservoirs. Different from dry gas reservoir, a liquid hydrocarbon phase called condensate is formed when the reservoir pressure around production wells drops below the dew point pressure in gas condensate formations. The liquid accumulates and occupies the pore space that otherwise would be available for gas flow, impeding the gas flow.

Supercritical carbon dioxide (SC-CO_2_) injection into gas reservoirs is considered to be a promising technology that will bring long-term mutual benefits for coupled increased productivity and CO_2_ geosequestration^3–12^. CO_2_ Enhanced Gas Recovery (EGR) has been studied in laboratory settings and in field-scale simulations for both tight and conventional gas reservoirs, but application in the field remains limited to a handful of cases^13–16^.

Excessive mixing is a key risk associated with CO_2_ EGR as it would result in undesirable contamination of the natural gas asset and/or early breakthrough of the injected CO_2_ at the production wells. This risk of excessive mixing of CO_2_ and natural gas within the reservoir has limited the practice of EGR. At present, rare literature reported the mix phenomena between CO_2_ and natural gas under reservoir conditions and clearly revealed under what kind of conditions natural gas is not easy to be mixed by CO_2_. Therefore, finding suitable gas injection conditions is the key to the promotion of EGR in suitable gas fields.

Although, CO_2_ flooding has been discussed in dry gas reservoirs, still, it has complex challenges in condensate reservoirs. One challenge is how to enhance condensate recovery; Another challenge is how to enhance condensate gas recovery. Previous research related to enhancing recovery of condensate gas field by injecting CO_2_ has until now focused on boosting condensate recovery. For this reason, the studied fluid of most reservoirs has been high-condensate content. Plenty of studies have been conducted to discuss the physical and chemical processes when CO_2_ contact with condensate liquid. These experimental data revealed the possibility of improving condensate by CO_2_ flooding and can be utilized to validate the numerical studies in modeling tools. For example, JJ Chaback *et al*.^17^, Ahmed *et al*.^18^, E. Shtepani^19^, and RM Ramharack^20^ observed the density and composition of CO_2_ in the process of retrograde evaporation of condensate oil through PVT experiment, and approved that CO_2_ can inhibit condensate precipitation and move condensate_._ Their studies indicated that the dew point drops, the liquid-volume percent (liquid saturation) decreases, the compressibility factor falls and the two-phase envelope diagram shrinks with the increase of CO_2_ composition. All these trends showed the positive impact of CO_2_ have on liquid recovery. Millán A E *et al*.^21^ and Al-Abri *et al*.^22,23^ quantitatively studied the condensate recovery and relative permeability with long core experiment and numerical simulation. Research showed that injecting SC-CO_2_ can decrease capillary instability, improve mobility ratio, delay the breakthrough time of gas injection and improve the condensate sweep efficiency. Heron GM *et al*.^24^ and Taheri A *et al*.^25^ carried out experimental study of injecting CO_2_, N_2_ and dry gas into fractured gas condensate reservoir. They came to conclusions opposed to each other.

However, there has been little investigation on how CO_2_ mix with condensate gas and whether CO_2_ can improve the recovery of condensate gas, despite these numerous studies on mitigating condensate precipitation and enhancing oil recovery by CO_2_ flooding. In fact, condensate gas reservoirs produce both condensate gas and condensate, and both have high profits. In order to elucidate the possibility of CO_2_ EGR in condensate gas reserovirs, a series of experiments and model were used to analyze mixing procedure when CO_2_ contact with condensate gas. The focus of this research is to demonstrate under what kind of conditions in terms of pressure, temperature and CO_2_ concentration, CO_2_ and condensate gas are not easily mixed into one phase.

## Experiment

### Experiment materials

The condensate gas from Well YK1, located at north of China is composed of 87.368% methane, 3.570% ethane and 0.718% propane etc. CO_2_ has purity up to 99.95%. The physical properties of condensate gas are listed in Table 2. The experimental conditions are that in the YKL gas reservoir (at 43.5 MPa and 132.18 °C).

**Table 2:** Summary of YKL condensate gas used in the experiments.

Component	Composition mol%	component	Composition mol%
CO_2_	1.290	nC_5_	0.156
N_2_	3.389	C_6_	0.504
C_1_	87.368	C_7_	0.677
C_2_	3.570	C_8_	0.429
C_3_	0.718	C_9_	0.390
iC_4_	0.161	C_10_	0.295
nC_4_	0.212	C_11+_	0.694
iC_5_	0.146		
C_11+_ molecular		199.5563	
C_11+_ relative density		0.8429	
Formation Volume Factor		1.0862	
Oil density (0.101 MPa, 20 °C)		0.7769 g/cm^3^	
Condensate pressure		15.00 MPa	
Formation pressure(MPa)		43.5	
Temperature (°C)		132.18	
Dew pressure (MPa)		37.62	

### Experimental apparatus

**Figure 1:**
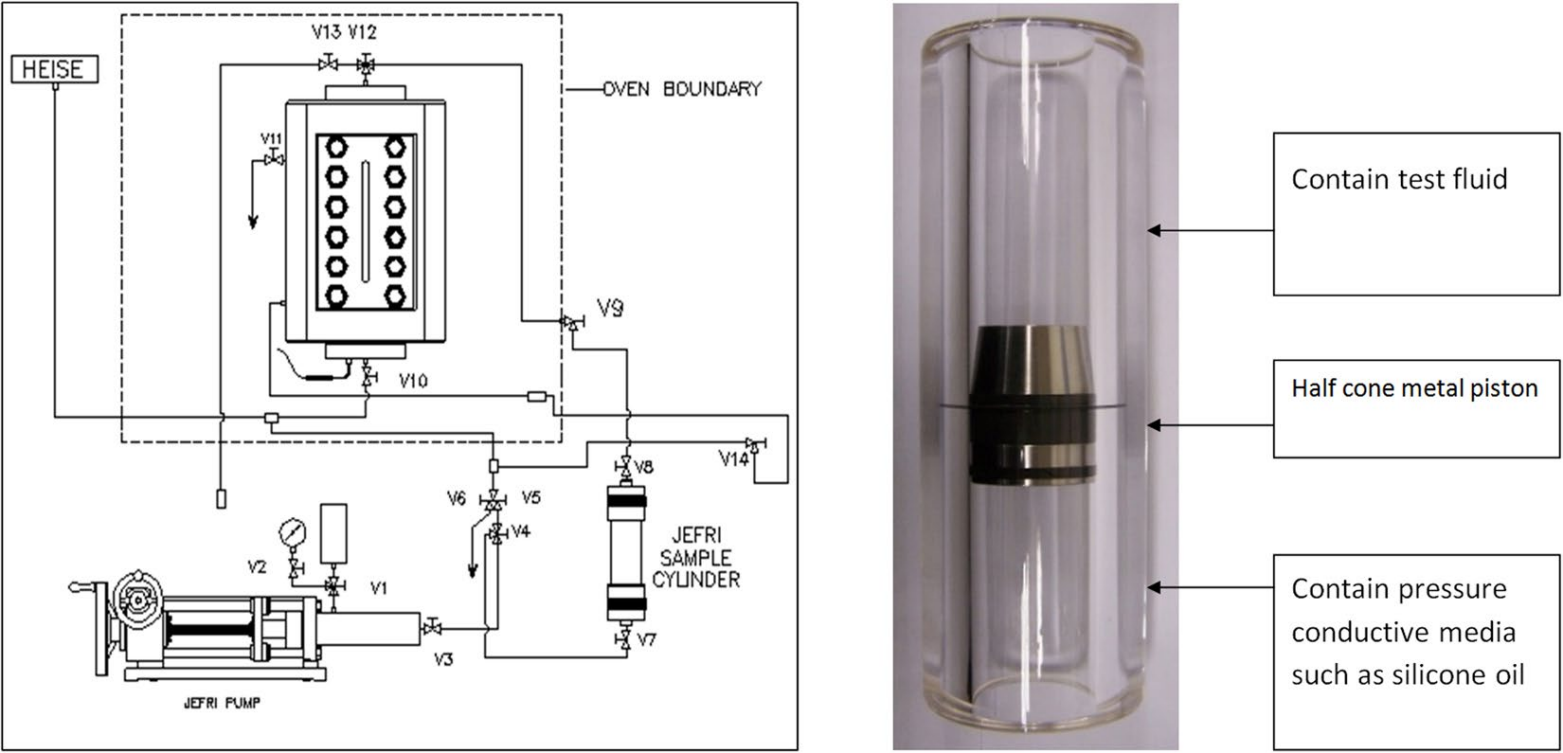
Schematic diagram of the experimental apparatus. (**a**) Is the overall schematic diagram of the experimental apparatus; (**b**) is transparent cylinder sapphire cell.

To observe and calibrate how CO_2_ mix with condensate gas, we set up a non-equilibrium experiment with high-pressure and high-temperature laboratory. A schematic of the experimental setup is presented in Fig. 1. A mercury-free DBR (Donald B. Robinson Company) PVT cell was used to study the phase behavior properties of the fluid samples. A transparent cylinder sapphire cell with a volume of approximately 150 ml and a maximum working pressure of 70 MPa at 200 °C was one of the most important parts of the experimental system. A half cone piston in cylinder cell can closely calibrate the fluid change in the experiment. The test fluid sample was placed in the cell by pushing a Ruska pump connected to the top of the cell. The volume of the injected sample was monitored by a cathetometer. Incandescent lamp, which was filled with hydrogen and N_2_, was placed at the opposite side of the sapphire cell to observe the change of the transmittance of the system. Fluid phase change in PVT cell was recorded via the computer video acquisition camera.

Before the experiments, the sapphire cell was dismounted from the apparatus, washed with distilled water and dried, and then installed back into the apparatus. Subsequently, the cell was evacuated to ensure the absence of air. The desired temperature was set through the temperature chamber. After that, the right amount of the sample that was equilibrated without condensate was prepared to be injected into the sapphire cell at desired conditions of pressure and temperature.

### Non-equilibrium phase measurement of CO_2_ mixed with condensate gas

Usually, it is difficult to survey the mixing process of CO_2_ and condensate gas in experiments through visual observation, because CO_2_ and condensate gas are both transparent. Inspired from the critical opalescence of CO_2_, critical opalescence is used to distinguish CO_2_ from condensate gas in these experiments. Critical opalescence is a striking light scattering phenomenon, which was elegantly explained by Einstein. In the critical region the light scattering is so large that the substance appears milky white in reflected light and brownish dark in transmitted light. The phenomenon arises from the large fluctuations in the critical region of the substance^26^. CO_2_ critical phenomenon around the critical temperature and pressure was reported in many literatures^27–29^. The critical temperature is 31 °C and critical pressure is 7.53 Mpa. However, the pressure and temperature of reservoir conditions are much higher than the critical pressure and critical temperature of CO_2_. No reports were mentioned about opalescence or density fluctuation of CO_2_ existed in high temperature and high pressure, which is far away from critical pressure and temperature.

The non-equilibrium phase measurements are aimed at finding the existence conditions of opalescence of CO_2_ over a wide range of temperatures and pressure, and characterizing the mix characteristic of CO_2_ and condensate gas at specific pressures and temperatures.

In the experiment, starting from a high pressure, it is gradually depressurized in the sapphire cell to observe physical changes through the glass window into the cell. Three different tests conducted: (1) Pressure and temperature search to find the opalescence of CO_2_ existence conditions; (2) Characterization of CO_2_ mixing with condensate gas; (3) Diffusion test of the system of condensate gas and SC-CO_2_ with high temperature.

#### Pressure and temperature search to find the opalescence of CO_2_ existence conditions

When the temperature was kept constant, the pressure search method was used to determine the CO_2_ opalescence existence conditions. The first step was to pump CO_2_ into sapphire cell at reservoir temperature (132.18 °C) and pressure (43.5 MPa). Then, pressure reduced from 43.5 MPa to 18 MPa, and CO_2_ status in sapphire cell was recorded every 3 minutes. To confirm the accuracy of the onset and end of the pressure of appearance and disappearance of opalescence CO_2_, three repetitive tests by increasing and decreasing the pressure around the appearance and disappearance of opalescence CO_2_ were preformed. Similar to the pressure search method, the temperature search method was used.

#### Characterization of CO_2_ mixing with condensate gas

Based on the pressure and temperature range at which CO_2_ displayed opalescence phase behavior, the pressure and temperature used to test the mixing of CO_2_ and condensate gas were selected. Then at specific temperature and pressure, injecting condensate gas from the top of sapphire cell steadily, took photos of the SC-CO_2_-condensate gas every five seconds from the visualization window.

#### Diffusion test of the system of condensate gas and SC-CO_2_ with high temperature

One group of temperature and pressure where CO_2_ appear opalescence phenomena were chosen. After the system standing for 30 minute, gas was discharge from sapphire cell with no pressure fluctuation. And their composition of different parts was measured with cathetometer.

## Results and Discussion

### Experiment Results

#### Pressure and temperature search to find the opalescence of CO_2_ existence conditions

**Figure 2:**
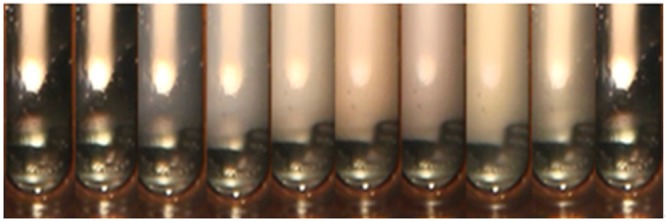
CO_2_ in PVT cell with vertical direction (132.18 °C).

Obvious phase changes of CO_2_ was recorded when pressure drops from 25 MPa to18MPa at 132.18 °C(Fig. 2).

Figure 2(1) and (2) shows that under the temperature and pressure of the gas reservoir (132.18 °C 43.5Mpa), the initial state of CO_2_ behaves like fluid with high density and stable physical properties. It is a kind of “liquid-like” supercritical fluid with the same light transmittance in the whole system. When the pressure drops to 25 MPa, CO_2_ opalescence appears in reservoir temperature for the first time (Fig. 2(3)). When the pressure drops continually, CO_2_ continues presenting opalescence phenomena in sapphire cell until 18Mpa (from Fig. 2(3) to (9)). When the pressure reaches to 18 MPa, opalescence phenomenon disappears and light transmittance increases significantly (Fig. 2(10)).

**Figure 3:**
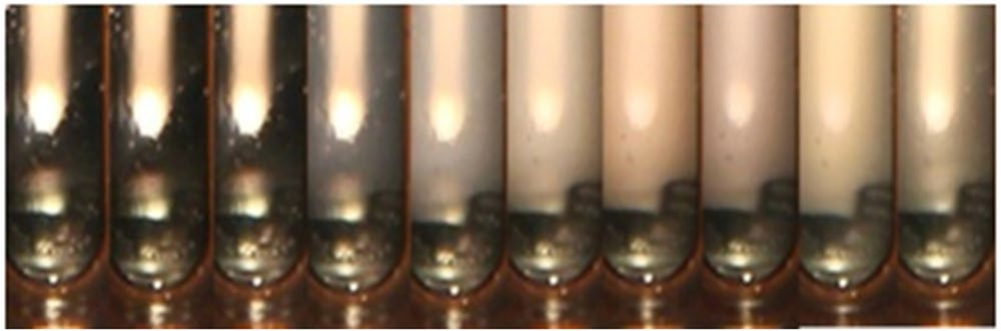
CO_2_ in PVT cell with vertical direction (30 MPa).

Similar phase changes of CO_2_ was recorded when temperature drops from 132 °C to 8 °C at 30 MPa (Fig. 3).

#### Distribution characterization of CO_2_ mixed with condensate gas

The pressures to test the mixing of CO_2_ and condensate gas were selected based on above pressure search experiments. 25Mpa was chosen as experiment pressure for mixing CO_2_ and condensate gas. Keep the temperature of this system in 132.18 °C. Injecting condensate gas from the top of sapphire cell containing CO_2_ steadily, photographs of SC-CO_2_-condensate gas from the visualization window were recorded very five seconds.

**Figure 4:**
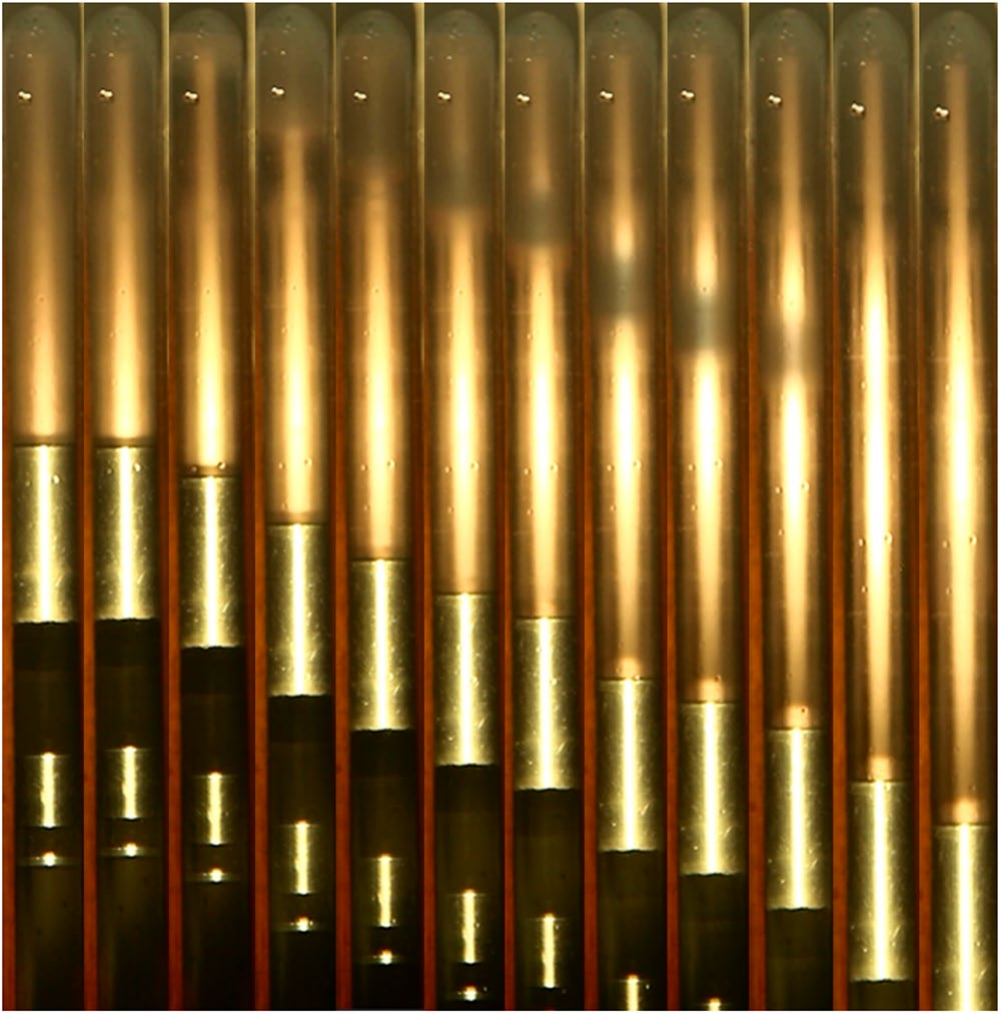
Unequilirium experiment of CO_2_ and condensate gas graph in vertical direction (from left to right condensate gas injected from above continually at 25 MPa 132. 18 °C).

The unequilirium experiment of CO_2_ and condensate gas snapshots revealed that obvious phase behavior change between CO_2_ and condensate gas at 25Mpa and 132.18 °C (Fig. 4). First the pure CO_2_ in the PVT cell, with weak background light source, was chosen as the basic condition before injecting condensate gas (Fig. 4(1)). At this point, CO_2_ stays in opalescence state with high density. After starting to inject condensate gas from the top of PVT cell, condensate gas looks like bright light appears at the top of CO_2_, which shows injected condensate gas has good transmittance. There is no obvious difference of the phase and physical characteristic between condensate gas and supercritical CO_2_ (Fig. 4(2 and 3)). After keeping injecting condensate gas from the top of PVT cell, there is convection diffusion and mass transfer between condensate gas and CO_2_. As condensate gas was injected from the top continually, the layer between two fluids with dark color appears. Similar to the phenomena near critical region reported in literature^27^, even temperature and pressure far away from critical point, these domains are dynamic and fluctuate strongly when the layer is between two fluids; we capture them immobilized during the transfer process(Fig. 4(4 and 5)). After keeping injecting condensate gas from the top of PVT cell, the layer between CO_2_ and condensate gas becomes clear (Fig. 4(6~10)). Continuing injecting condensate gas from the top of sapphire cell, the layer between CO_2_ and condensate gas shrinks and then contracts to a bright yellow opaque. There exists transparent uniform condensate gas on the top and transparent supercritical CO_2_ phase at the bottom (Fig. 4(11 and 12)).

Similar phenomena were also recorded at 22 MPa and 20 MPa.

The temperatures to test the mixing of CO_2_ and condensate gas were selected based on above temperature search experiments. Similar phenomena about mixing of CO_2_ and condensate gas were also recorded at 100 °C, 50 °C and 30 °C.

The experimental data with the onset and end pressure of layer appearance with different temperature, pressure and CO_2_ mole percentage were listed in Tables 3 and 4. When temperature drops from 132 °C to 30 °C, there is more possibility of the layer existed between CO_2_ and condensate gas. From 132.18 °C to 30 °C, there is a gradual declined reduction in the end pressure of layer, reaching a figure of 7.5 Mpa.

**Table 3:** The onset pressure of layer appearance (MPa).

CO_2_(mol%)	10	20	30	40	50	60	70	80	90
TEMP (°C)									
132.18	—	—	28.79	25.47	24.97	20.77	—	—	—
100		34.56	30.76	29.74	25.43	22.81	20.14	—	—
50		36.33	32.49	28.91	25.48	23.46	18.81	15.51	11.29
30	38.70	38	36.05	28.00	24.37	20.58	17.81	12.97	10.50

**Table 4:** The end pressure of layer (MPa).

CO_2_ (mol%)	10	20	30	40	50	60	70	80	90
TEMP (°C)									
132.18	—	—	25.46	19	18	20	—	—	—
100	—	33.56	20	14	12	14	19	—	—
50	—	18	12.29	10.5	9	10	10	11	12.29
30	16	11	8.89	7.5	7.5	7.5	7.5	7.5	7.5

#### Diffusion test of the system of condensate gas and SC-CO_2_ with high temperature

**Figure 5:**
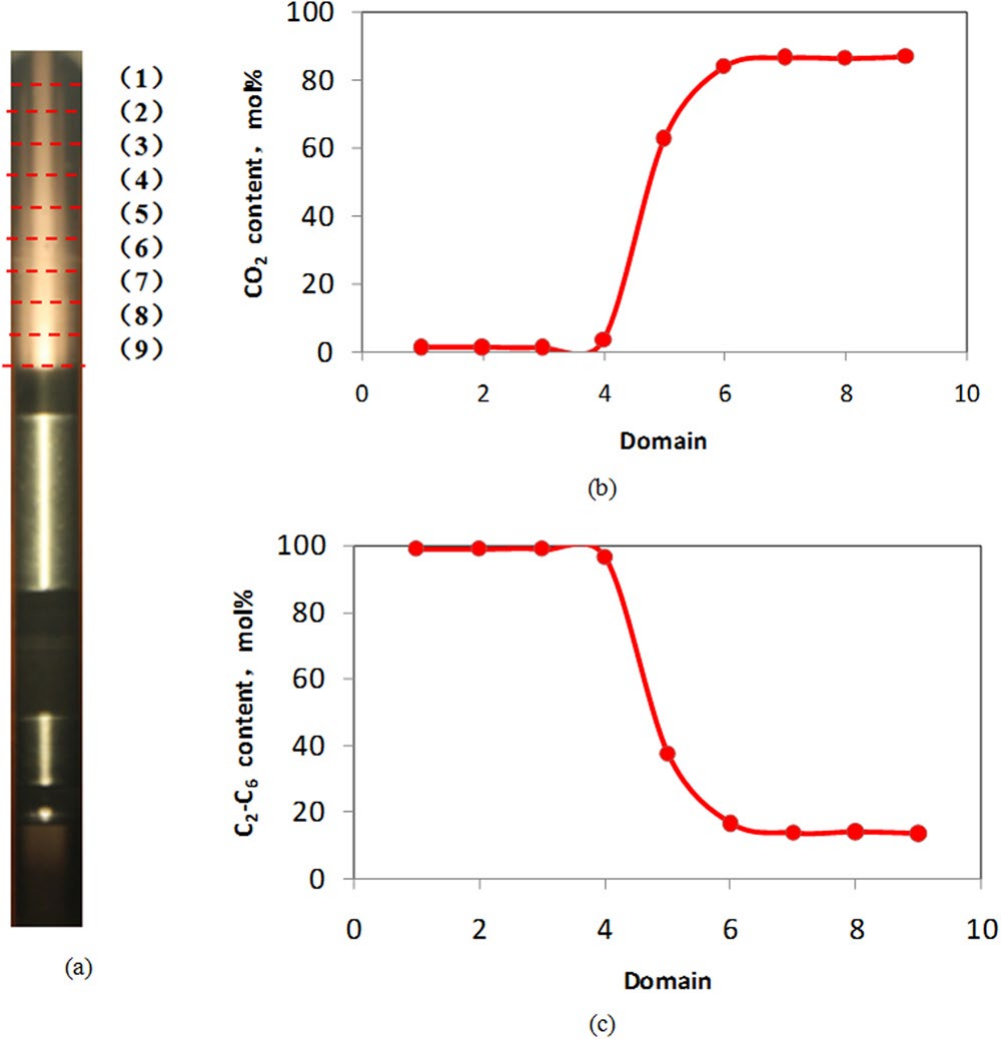
Composition measure in PVT cell. (**a**) Snapshot of mixing fluid in PVT cell that is divided into 10 equal division subdomains. (**b**) CO_2_ content of different domains (**c**) C_2–6_ content of different domains.

To perform a detailed assessment of the spatial distribution of CO_2_ and condensate gas and discuss interface property, the diffusion test was carried out with the composition analysis of 10 equal division subdomains based on pressure search experiment. Fig. 5 and Table 5 shows the composition of different subdomains in Sapphire cell containing condensate gas and CO_2_ when pressure is 25 MPa and temperature is 132.18 °C, after mix experiment was stood still for 30 minutes. The gas was discharged and tested with cathetometer. Composition shows that domain 1–3 is primarily composed of condensate gas. Domain 4 to 6 see a dramatic rise of CO_2_ content rise and changed from 3.519 to 86%; a considerable decrease of the C_2_~C_6_ content occurs from 5.39% to 1.95%; as for the domain 6 to 9, the content of compositions remain still. Thus, compared with the composition of different domains, the interface between condensate gas and CO_2_ locate at domain 4–5, which means that the composition of interface may be a compound of CO_2_ and condensate gas. Based on the definition^30^, the layer between condensate gas and CO_2_ is also called interface. Aforementioned conclusion needs further investigation.

**Table 5:** The composition of gas condensate and supercritical CO_2_ at different domains.

Domain	(1)	(2)	(3)	(4)	(5)	(6)	(7)	(8)	(9)
Composition (mol%)									
CO_2_	1.291	1.291	1.291	3.549	62.65	83.652	86.432	86.126	86.659
N_2_	3.477	3.477	3.477	3.218	1.82	1.686	1.475	1.823	1.534
C_1_	89.624	89.624	89.624	87.842	32.957	12.718	10.312	10.218	9.951
C_2_	3.662	3.662	3.662	3.688	1.747	1.274	1.204	1.192	1.201
C_3_	0.737	0.737	0.737	1.076	0.539	0.389	0.373	0.372	0.379
IC_4_	0.165	0.165	0.165	0.131	0.059	0.051	0.046	0.046	0.049
NC_4_	0.217	0.217	0.217	0.29	0.12	0.117	0.113	0.113	0.115
IC_5_	0.150	0.150	0.150	0.012	0.011	0.009	0.008	0.008	0.009
NC_5_	0.160	0.160	0.160	0.066	0.028	0.031	0.03	0.03	0.03
C_6_	0.517	0.517	0.517	0.127	0.069	0.074	0.007	0.073	0.073

### Interface property analysis

#### Interface property at 132.18 °C

The onset pressure of layer/interface appearance: In the experiment, when the temperature is 132.18 °C, the onset pressure of interface appearance is 25 MPa when 50 mol% of CO_2_ mixes with of 50 mol% condensate gas. The PR equation of state, which was fitted to experiment data, was used for two-phase flashing calculation under different experimental conditions. The calculation results are shown in Table 6. From the results, it can be seen that when the pressure is 25 MPa, the system with 50 mol% of condensate gas and 50 mol% of CO_2_ begin be condensed, which corresponds to the onset pressure of the interface. Therefore, it is believed that there may be an interface when condensate is condensed in CO_2_-condensate gas system.

**Table 6:** Two-phase flash with different pressure (50% CO_2_, 50% Condensate Gas Temperature 132.18 °C).

Pressure, MPa	Oil Viscosity cp	Gas Viscosity cp	Gas Z-factor	Oil Z-factor	IFT dyn/cm	Gas density	Oil density
43.5		0.0441	0.9872			426.89	
42		0.043	0.9752			417.239	
40		0.0416	0.9596			403.82	
38		0.0401	0.9446			389.72	
36.07		0.0386	0.9308			375.43	
35		0.0378	0.9234			367.20	
30		0.0339	0.8925			325.64	
25		0.0300	0.8697			278.49	
24.97	0.296	0.0292	0.8663	1.2139	2.8472	268.03	786.78
24	0.298	0.0284	0.8639	1.1802	3.1018	257.64	786.90
23	0.300	0.0276	0.8619	1.1442	3.387	246.73	786.96
22	0.301	0.0268	0.8606	1.1069	3.696	235.65	786.93
21	0.303	0.0261	0.8598	1.0683	4.0303	224.43	786.82
20	0.304	0.0254	0.8597	1.0283	4.3914	213.06	786.61
19	0.306	0.0247	0.8603	0.987	4.7808	201.56	786.31
18	0.307	0.0240	0.8615	0.9444	5.2002	189.95	785.92
17	0.308	0.0234	0.8636	0.9004	5.6511	178.25	785.446
16	0.309	0.0228	0.8663	0.8552	6.1352	166.49	784.9
15	0.310	0.0222	0.8699	0.8088	6.6542	154.68	784.31
14	0.311	0.0217	0.8742	0.7613	7.2102	142.85	783.73
13	0.312	0.0212	0.8794	0.7128	7.8057	131.04	783.21
12	0.314	0.0208	0.8853	0.6633	8.4436	119.27	782.84
11	0.315	0.0204	0.8921	0.6131	9.1275	107.57	782.74
10	0.318	0.0200	0.8996	0.5621	9.8618	95.97	783.07
9	0.321	0.0196	0.908	0.5104	10.6518	84.51	784.02
8	0.325	0.0193	0.9171	0.458	11.5041	73.2	785.81
7	0.33	0.0191	0.9269	0.4047	12.4267	62.08	788.77
6	0.337	0.0188	0.9374	0.35	13.4291	51.16	793.26
5	0.345	0.0188	0.9486	0.2934	14.5228	40.45	799.786

The end pressure of layer/interface: However, with the decrease of pressure, the condensate content in the interface rise and the interfacial tension between CO_2_ and condensate grows, and phase separation occurs. When the condensate in the interface is precipitated continuously, the interface disappears gradually due to the influence of gravity differentiation.

In order to prove above idea, numerical simulation of slimtube in CO_2_ drive condensate under different pressure was carried out to analyze the interfacial tension between CO_2_ content and condensate.

#### Analysis of interfacial tension in rich CO_2_ condensate

**Figure 6:**
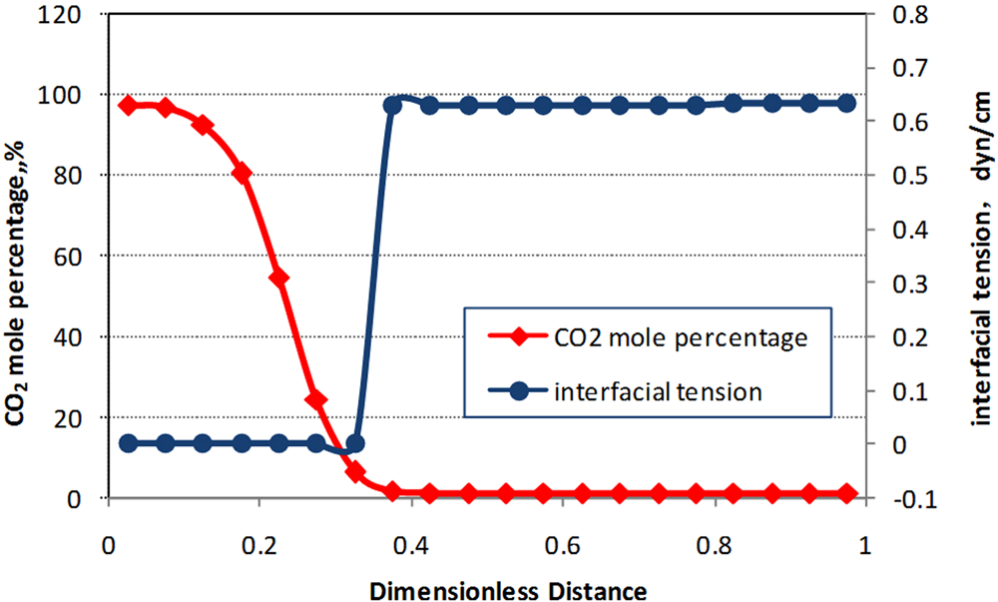
The simulated CO_2_ mole percentage and Interfacial tension (25 MPa 132.18 °C). Dimensionless distance 0 represent injector and Dimensionless distance 1 represent producer.

**Figure 7:**
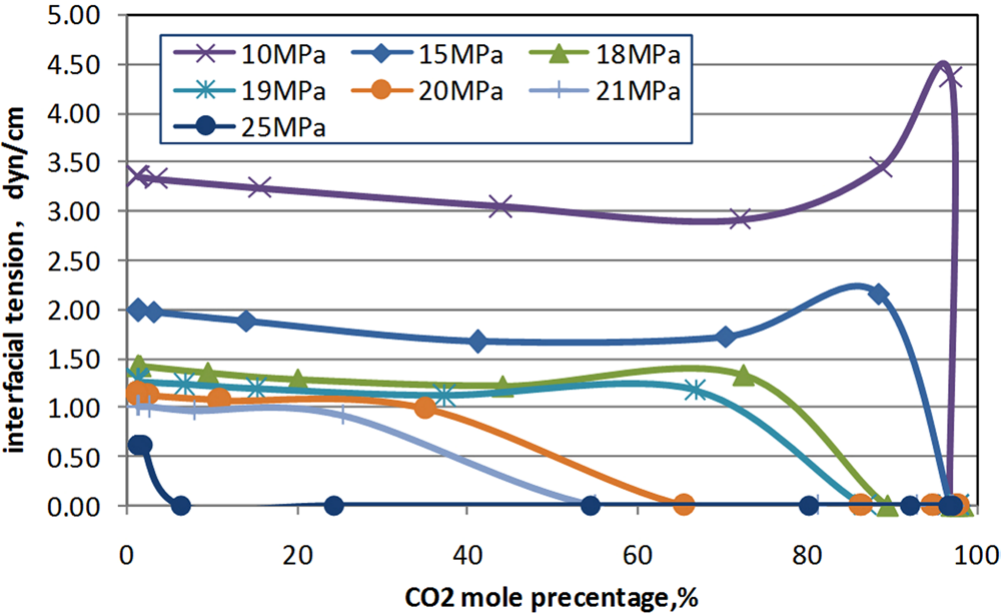
The simulated Interfacial tension with CO_2_ mole percentage and pressure.

The CO_2_ mole percentage and interfacial tension were plotted for driving simulation of condensate. Dimensionless distance 0.375 is the CO_2_ displacement front of in the displacement process of condensate, shown in Fig. 6. At the displacement front of CO_2_, there is low interfacial tension between CO_2_ and condensate, which is about 0.6 dyn/cm, when the pressure is 25 MPa. With the pressure decreasing, the interfacial tension between CO_2_ and condensate increases gradually (Fig. 7). Therefore, when the temperature is 132.18 °C and the pressure is 25 MPa, CO_2_ can mix with condensate and forms interface. But with the decrease of pressure, condensate in the interface is separated, and the interface begins to be unstable.

#### Density analysis

**Figure 8:**
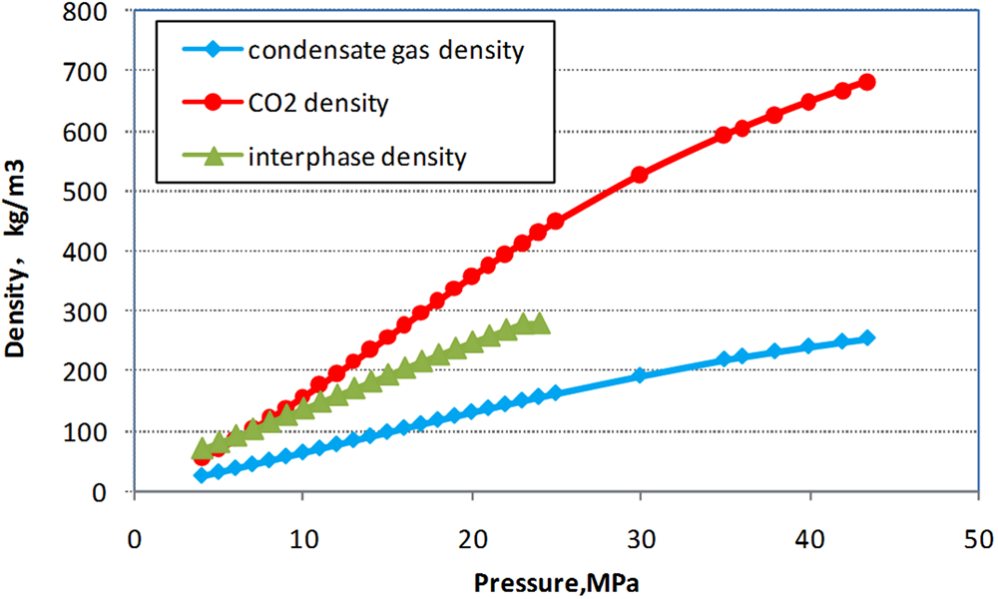
The density of three phases at different pressure.

The density of interface was calculated based on molar percentage of the condensate and CO_2_. At 132 °C, the density of supercritical CO_2_, condensate gas and interface were plotted in the Fig. 8. When the pressure is 24.97 MPa, there is a stable density difference among three phases. With the pressure decreasing, the density of interface is closer to the density of supercritical CO_2_. When the pressure is below 10 MPa, the densities of interface and condensate gas are exactly the same. Thus, in order to form a stable interface, it is considered that there is a certain density difference between the three phases.

### Modeling Results

Obtaining this experiment conditions including pressure, temperature and CO_2_ concentration which can yield interface phenomena is a very labor intensive and time consuming process, especially when the components differ greatly from different gas fields. A possible solution to this problem is to develop a new approach to describe experimental data that would allow construction phase transition interface out of a limited data set. Such approach will make it possible to obtain not only reliable information on properties of mixtures, but also data on composition.

Based on interface property analysis, novel mathematical model to model the interface phenomena during CO_2_ mixing with condensate gas was established, in order to find the region where the conditions in terms of pressure, temperature and CO_2_ concentration can yield VLV equilibrium containing interface, which is presented in Appendices.

#### Reliability of VLV calculation

The accuracy of the calculations was evaluated by the absolute average relative deviation (AARD) defined as follows,

1 $${\bf{AARD}}=\frac{{\bf{100}}}{{\bf{N}}}\sum _{{\bf{i}}={\bf{1}}}^{{\bf{N}}}\,\frac{|{{\bf{x}}}_{{\bf{i}}}^{{\bf{calc}}.}-{{\bf{x}}}_{{\bf{i}}}^{\exp {\boldsymbol{.}}}|}{{{\bf{x}}}_{{\bf{i}}}^{\exp {\boldsymbol{.}}}}$$

**Figure 9:**
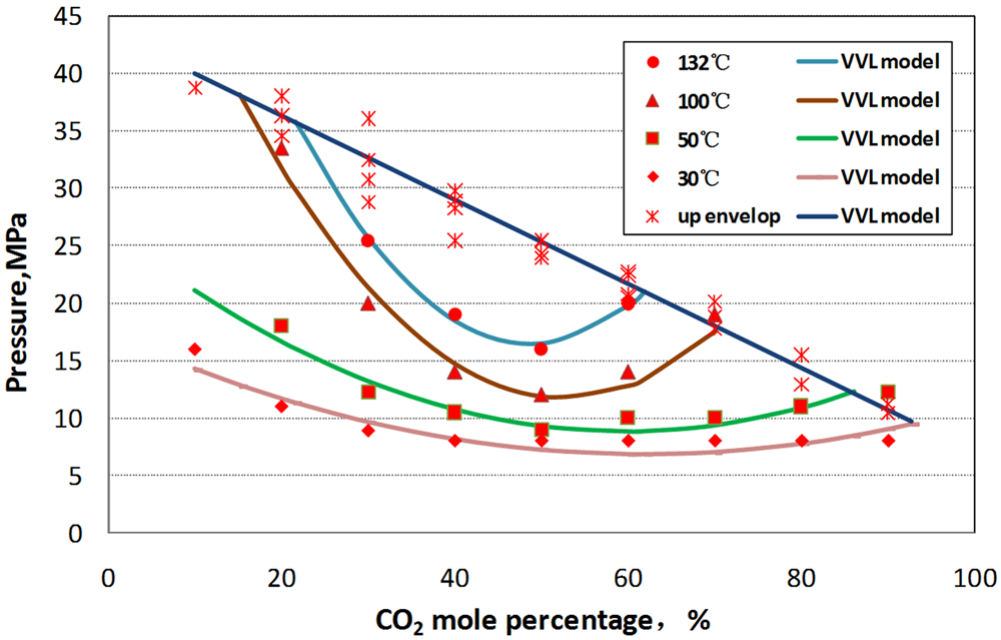
Comparison of Pressure of interface envelop of experiments and VLV model at 132 °C over a CO_2_ mole percentage range of 10–95%.

As shown in Fig. 9, experimental pressures at which interface appear and disappear with different CO_2_ mole percentage at 30 °C, 50 °C, 100 °C and 132 °C were in well agreement with that calculated by the VLV model with AARD = 5.41%, indicating that the VLV model in present work was reliable for interface envelop calculation.

#### PTX phase diagram of interface

**Figure 10:**
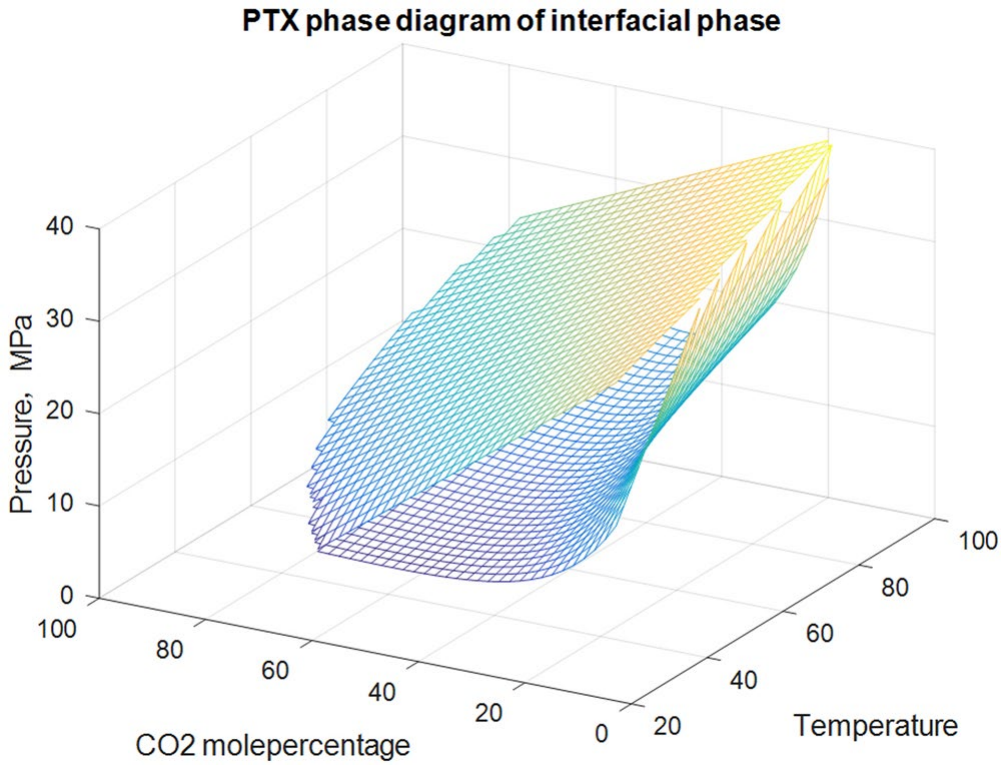
PTX phase diagram of interface.

**Figure 11:**
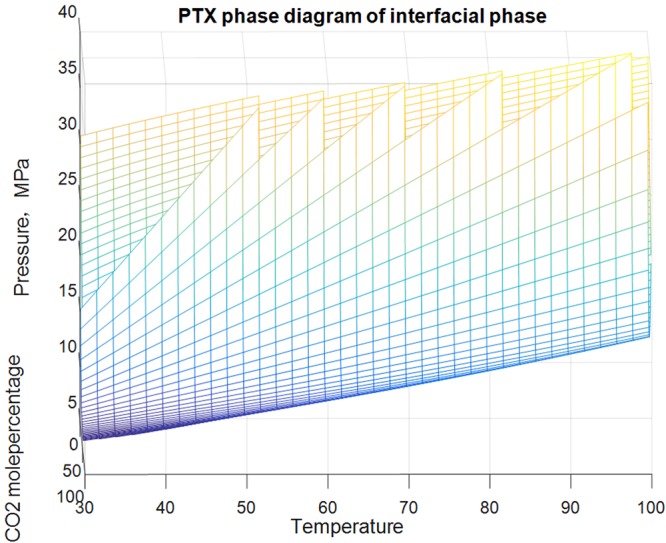
PT phase diagram of interface.

The PTX phase diagram of interface was drawn in Fig. 10. Figure 10 shows that the interface envelops looked like J column or “stomach”. At the temperatures above CO_2_ critical temperature (31 °C), interface occurs when CO_2_ contact with condensate gas (see Fig. 11). With the temperature rising, the up pressure and low pressure envelope of the interface increase gradually.

**Figure 12:**
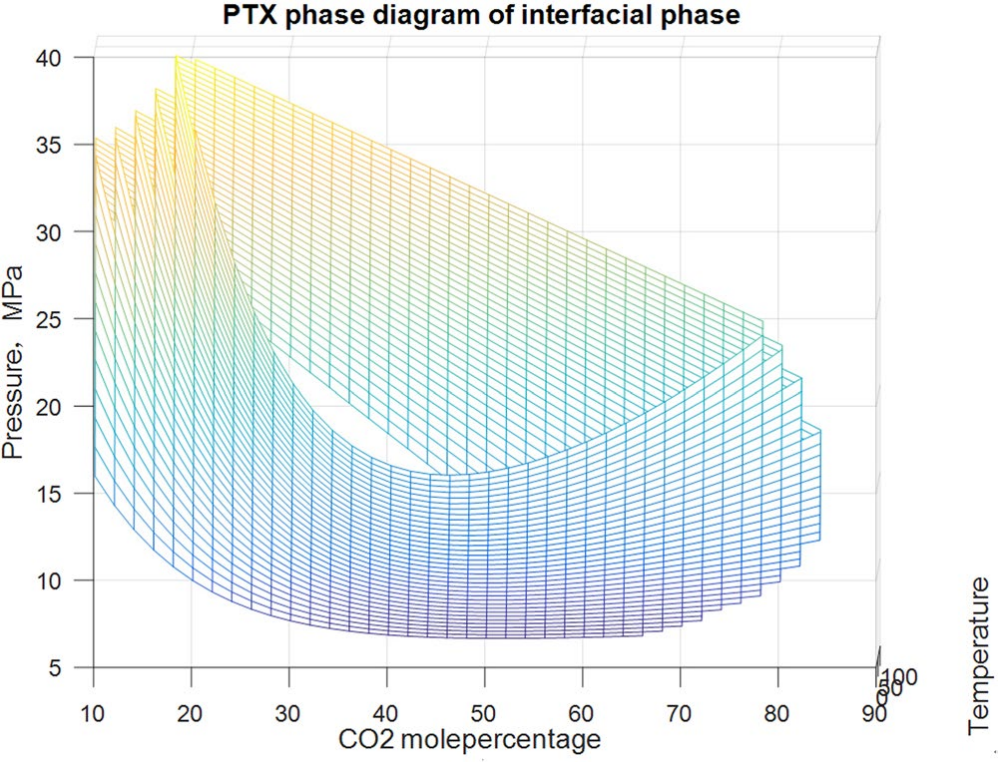
PX phase diagram of interface.

Figure 12 illustrates that when CO_2_, the content of which is between 10% and 82%, mix with condensate gas, the system shows interface characteristics in a certain pressure range; when the content of CO_2_ is between 40 and 50 mol%, a wide pressure range of interface appears in the system.

## Conclusion

The following conclusions can be drawn from the current study:

- Measures show opalescence or density fluctuation of CO_2_ in high temperature and high pressure. Opalescence phenomena of CO_2_ in high temperature and high pressure was used to observe the mix process of CO_2_ and condensate gas, which shows there is interface when CO_2_ mix with condensate gas at high (reservoir) temperature.
- Thermodynamic characteristic analyses indicate the onset pressure of interface appearance between CO_2_ and condensate gas is around dew pressure in the CO_2_-condensate system. With the pressure dropping, the condensate in the interface is precipitated continuously. The end pressure of interface appearance is related to gravity differentiation because of increase of the interfacial tension and phase separation between CO_2_ and condensate.
- The multiphase thermodynamic model for condensate-CO_2_ system in this paper can truthfully reflect the interface behavior during CO_2_ injecting condensate gas over a wide range of temperatures and pressures, and calculation results of which are close to the experimental data.
- Our study provides the possibility to predict the enhanced condensate gas recovery with CO_2_ injection with novel trial.

## Electronic supplementary material

Appendix A
