# Supplementary material for: The Vapour-Vapour Interface Observation and Appraisement of a Gas-Condensate/Supercritical CO2 System
Source: Sci Rep. 2018 Sep 28;8:14510. doi: 10.1038/s41598-018-32622-9 (PMC6162297; doi:10.1038/s41598-018-32622-9)
Supplement: Supplementary file 1 — Appendix A [file 41598_2018_32622_MOESM1_ESM.docx]

The Vapour-Vapour Interface Observation and Appraisement of a Gas-Condensate/ Supercritical CO_2_ System

Ying Jia^1*^, Yunqing Shi^1^, Lei Huang^2^, Jin Yan^1^, Rongchen Zheng^1^ and Lei Sun^3^

Appendix A- phase behavior model considering interface between condensate gas and SC-CO_2_

***A.1 Representation of interfacial phase***

The interface is represented as a compound of CO_2_ and condensate. The fugacity of component i in interface ($j=l$) and condensate gas ($j=v2$) are:

$\ln f_{\mathrm{ij}}=\ln f_{\mathrm{ij}}^{0}+\frac{s_{i}b_{i}p}{\mathrm{RT}};i=1\ldots\ldots n_{c};j=v2,l$ (a1)

Where,

$\ln f_{\mathrm{ij}}$ fugacity of component i in phase j with volume shift, MPa；$\ln f_{\mathrm{ij}}^{0}$ fugacity of component i in phase j without volume shift, MPa；$s_{i}$ dimensionless volume shift

and b, the EOS “b” parameter for component *i*，i.e.

$b_{i}=\frac{\Omega_{b}RT_{ci}}{p_{ci}}$ (a2)

Where,

$p_{\mathrm{ci}}$ critical pressure of component i, MPa; $T_{\mathrm{ci}}$ critical temperature of component i, MPa; $\Omega_{b}$ dimensionless EOS parameter

The molar volume of phase j with volume shift is:

$v_{j}=v_{j}^{0}+\sum_{i=1}^{n_{c}} y_{\mathrm{ij}}s_{i}b_{i};j=v2,l$ (a3)

Where, $v_{j}^{0}$is the EOS molar volume without volume shift. The volume shift parameter was first introduced by Peneloux et al. to improve liquid density prediction. It was found that this parameter is also important in modeling the onset and end pressure of interfacial phase.

The Peng-Robinson EOS is used for all calculation in this paper.

***A.2 Components of three phases***

In a mixture of n_c_ components, let the interface is a compound of CO_2_ and condensate, *v*1 phase is pure CO_2_; *v*2 phase is condensate gas phase; and *l* is interface. When the three phases coexist, the following thermodynamic equilibrium equations are satisfied:

$i=1\ldots\ldots n_{c}$ (a4)

$i=1\ldots\ldots n_{c}$ (a5)

In this work, the interaction coefficients between hydrocarbons are calculated from:

$\text{d}_{\text{ik}}=1-\left(\frac{2\cdot\text{v}_{\text{ci}}^{1\text{/}6}\cdot\text{v}_{\text{ck}}^{1\text{/}6}}{\text{v}_{\text{ci}}^{1\text{/}3}+\text{v}_{\text{ck}}^{1\text{/}3}}\right)^{\text{e}}$ (a6)

Where,

$\text{d}_{\text{ik}}$ interaction coefficients between component i and k , dimensionless; $e$ adjustable parameter, dimensionless; $\text{v}_{\text{ci}}$ critical volume of component i, m^3^/kmol; $\text{v}_{\text{ck}}$ critical volume of component k, m^3^/kmol

With larger interaction coefficients with CO_2_, the interface component becomes more “incompatible” or unstable with CO_2_ and then condensate tends to separate with the mount of condensate increase. Although *l* called interface, the amount of interface that coexist in the system is govern by Equation (a4,a5).

Interaction coefficients between components are important parameters for phase behavior predictions.

***A.3 Estimation of Model Parameters***

At a given temperature T, the parameters associated with the interface are

The fugacity of interface $\text{ln}f_{il}^{*}$at a reference pressure p*.

The volume shift parameter of the interface.

The molar volume of interface

The Interaction coefficients between CO_2_ and condensate in interface.

***A.4 Flash Calculations with interface***

***A 4.1 Stability Test***

Given a pressure p, a temperature T, and a mixture with global composition zi (i=1,…,nc), flash calculations determine the phase molar fraction and composition by solving Equation (a4,a5). The complexity of multiphase flash calculations is due to the fact that the number of phases in equilibrium is not known a priori. A stage wise method for performing multiphase flash calculation with an EOS was used ^[1-7]^.

***A4.2 Flash calculations***

Flash calculations consist of determining the phase splits and phase compositions for a feed with moles *x*_i_ i=1,2,…,n_c_, at a given p and T. Instead of *x*_i_, the feed composition (global composition) can be specified. The following equations apply:

$x_{i}=x_{iv2}+x_{il}$ $i=1\ldots\ldots n_{c},i\neq\mathrm{CO}_{2}$ (a7)

$x_{i}=x_{iv1}+x_{iv2}+x_{il}$ $i=\mathrm{CO}_{2}$ (a8)

$x_{t}=\sum_{i=1}^{n_{c}} x_{i}$ (a9)

$z_{i}=\frac{x_{i}}{x_{t}}$ (a10)

Where,

$x_{ij}$ moles of component i in phase j; $x_{t}$ total moles in hydrocarbon phases Equation (a4,a5) form a system of (n_c_+1) equations that can be solved for $x_{iv2}$ $i=1\ldots\ldots n_{c}$ and $x_{l1}$ with Newton’s method. Because of the existence of multiple solutions, a good initial of solution is normally required to obtain convergence. The Quasi Newton Successive Substitution (QNSS) method was used.

In flash calculations, the equilibrium equations are often written in terms of equilibrium ratios (K-value). The three-phase equilibrium Equations (a4,a5) can be rewritten as:

$i=1\ldots\ldots n_{c}$ (a11)

$i=1\ldots\ldots n_{c}$ (a12)

$\ln k_{i}^{v2l}+\ln\varphi_{i}^{L}-\ln\varphi_{i}^{v2}=0$ (a13)

$k_{i}^{v1l}=\frac{x_{iv1}}{x_{i\text{l}}}=\frac{\varphi_{i}^{L}}{\varphi_{i}^{v1}}$ (a14)

$k_{i}^{v2l}=\frac{x_{iv2}}{x_{i\text{l}}}=\frac{\varphi_{i}^{L}}{\varphi_{i}^{v2}}$ $\mathbf{i}\boldsymbol{\neq}\mathbf{CO}_{\mathbf{2}}$ (a15)

$k_{i}^{v2l}=\frac{1}{x_{co2v2}}=\frac{\varphi_{i}^{L}}{\varphi_{co2}^{v2}}$ $i=CO_{2}$ (a16)

VLV three phase equilibrium model is established as follows,

$\sum_{i=1}^{n} x_{iv1}=\sum_{i=1}^{n} \frac{z_{i}k_{i}^{v1l}}{V_{1}\left(k_{i}^{v1l}-1\right)+V_{2}\left(k_{i}^{v2l}-1\right)+1}=1$ (a17)

$\sum_{i=1}^{n} x_{iv2}=\sum_{i=1}^{n} \frac{z_{i}k_{i}^{v2l}}{V_{1}\left(k_{i}^{v1l}-1\right)+V_{2}\left(k_{i}^{v2l}-1\right)+1}=1$ (a18)

$\sum_{i=1}^{n} x_{il}=\sum_{i=1}^{n} \frac{z_{i}}{V_{1}\left(k_{i}^{v1l}-1\right)+V_{2}\left(k_{i}^{v2l}-1\right)+1}=1$ (a19)

Where,$V_{1}$、$V_{2}$、$L$are mole fraction of pure CO_2_, condensate gas and interface, MPa.

In conjunction with the above equations, According to Equation (a18, a19), the following material balance equations can be derived:

$\left\{ \begin{matrix} \sum_{\boldsymbol{i=1}}^{\boldsymbol{N}} \frac{\left( \boldsymbol{k}_{\boldsymbol{i}}^{\boldsymbol{v}\boldsymbol{2}\boldsymbol{l}}\boldsymbol{-1} \right)\boldsymbol{z}_{\boldsymbol{i}}}{\boldsymbol{L}\left( \boldsymbol{k}_{\boldsymbol{i}}^{\boldsymbol{v}\boldsymbol{2}\boldsymbol{l}}\boldsymbol{-1} \right)\boldsymbol{+1-}\boldsymbol{V}_{\boldsymbol{1}}}\boldsymbol{,}\mathbf{i}\boldsymbol{\neq}\mathbf{CO}_{\mathbf{2}} \\ \boldsymbol{k}_{\boldsymbol{i}}^{\boldsymbol{v}\boldsymbol{1}\boldsymbol{l}}\boldsymbol{=}\frac{\boldsymbol{k}_{\boldsymbol{i}}^{\boldsymbol{v}\boldsymbol{2}\boldsymbol{l}}\left( \boldsymbol{z}_{\boldsymbol{co}\boldsymbol{2}}\boldsymbol{-}\boldsymbol{V}_{\boldsymbol{1}} \right)}{\boldsymbol{k}_{\boldsymbol{i}}^{\boldsymbol{v}\boldsymbol{2}\boldsymbol{l}}\left( \boldsymbol{1-}\boldsymbol{V}_{\boldsymbol{1}}\boldsymbol{-L} \right)\boldsymbol{-1}}\boldsymbol{,}\mathbf{i=}\mathbf{CO}_{\mathbf{2}} \end{matrix} \right.$ (a20)

QNSS method is used to obtain quadratic convergence.

Reference:

1. Michelsen, M.L. The isothermal flash problem. Part I. Stability. *Fluid Phase Equilibria* **9**,1-19 (1982).
2. Michelsen, M.L. The isothermal flash problem. Part II. Phase-split calculation. *Fluid Phase Equilibria* **9**(1):21-40 (1982).
3. Nelson, P.A. Rapid Phase Determination in Multiple-Phase Flash Calculations. *Computers & Chemical Engineering* **11**, 581-591 (1987).
4. Cañas-Marín, W.A. *et al.* Improved Two-Sided Tangent Plane Initialization and Two-Phase-Split Calculations. *Ind. Eng. Chem. Res.* **46**, 5429-5436 (2007).
5. Haugen, K.B. *et al.* Efficient and robust three-phase split computations. *AIChE J*.**57**,2555-2565 (2011).
6. Li, Z.D. & Firoozabadi, A. Initialization of phase fractions in Rachford-Rice equations for robust and efficient three-phase split calculation. *Fluid Phase Equilibria* **332**, 21-27 (2012).
7. Li, Z.D. and Firoozabadi, A. General Strategy for Stability Testing and Phase-Split Calculation in Two and Three Phases. *SPE Journal* **17**, 1096-1107 (2012).
